# Optimizing ultrasound molecular imaging of secreted frizzled related protein 2 expression in angiosarcoma

**DOI:** 10.1371/journal.pone.0174281

**Published:** 2017-03-23

**Authors:** James K. Tsuruta, Nicholas P. Schaub, Juan D. Rojas, Jason Streeter, Nancy Klauber-DeMore, Paul Dayton

**Affiliations:** 1 Joint Department of Biomedical Engineering, North Carolina State University, and The University of North Carolina at Chapel Hill, Chapel Hill, North Carolina, United States of America; 2 Department of Pediatrics, University of North Carolina at Chapel Hill, Chapel Hill, North Carolina, United States of America; 3 Department of Surgery, University of North Carolina at Chapel Hill, Chapel Hill, North Carolina, United States of America; 4 Department of Surgery, Medical College of South Carolina, Charleston, South Carolina, United States of America; 5 Division of Molecular Pharmaceutics, UNC Eshelman School of Pharmacy, Chapel Hill, North Carolina, United States of America; Emory University, UNITED STATES

## Abstract

Secreted frizzled related protein 2 (SFRP2) is a tumor endothelial marker expressed in angiosarcoma. Previously, we showed ultrasound molecular imaging with SFRP2-targeted contrast increased average video pixel intensity (VI) of angiosarcoma vessels by 2.2 ± 0.6 VI versus streptavidin contrast. We hypothesized that redesigning our contrast agents would increase imaging performance. Improved molecular imaging reagents were created by combining NeutrAvidin^™^-functionalized microbubbles with biotinylated SFRP2 or IgY control antibodies. When angiosarcoma tumors in nude mice reached 8 mm, time-intensity, antibody loading, and microbubble dose experiments optimized molecular imaging. 10 minutes after injection, the control-subtracted time-intensity curve (TIC) for SFRP2-targeted contrast reached a maximum, after subtracting the contribution of free-flowing contrast. SFRP2 antibody-targeted VI was greater when contrast was formulated with 10-fold molar excess of maleimide-activated NeutrAvidin^™^ versus 3-fold (4.5 ± 0.18 vs. 0.32 ± 0.15, VI ± SEM, 5 x 10^6^ dose, p < 0.001). Tumor vasculature returned greater average video pixel intensity using 5 x 10^7^ versus 5 x 10^6^ microbubbles (21.2 ± 2.5 vs. 4.5 ± 0.18, p = 0.0011). Specificity for tumor vasculature was confirmed by low VI for SFRP2-targeted, and control contrast in peri-tumoral vasculature (3.2 ± 0.52 vs. 1.6 ± 0.71, p = 0.92). After optimization, average video pixel intensity of tumor vasculature was 14.2 ± 3.0 VI units higher with SFRP2-targeted contrast versus IgY-targeted control (22.1 ± 2.5 vs. 7.9 ± 1.6, p < 0.001). After log decompression, 14.2 ΔVI was equal to ~70% higher signal, in arbitray acoustic units (AU), for SFRP2 versus IgY. This provided ~18- fold higher acoustic signal enhancement than provided previously by 2.2 ΔVI. Basing our targeted contrast on NeutrAvidin^™^-functionalized microbubbles, using IgY antibodies for our control contrast, and optimizing our imaging protocol significantly increased the SFRP2-specific signal returned from angiosarcoma vasculature, and may provide new opportunities for targeted molecular imaging.

## Introduction

Angiogenesis is the formation of new capillaries from existing microvasculature. There is a critical need for non-invasive imaging techniques that examine molecular events associated with the angiogenic process that could prove invaluable for improving specificity for diagnosing malignancies. One strategy for studying angiogenesis is ultrasound molecular imaging, which differs from traditional ultrasound imaging in that targeted contrast agents selectively adhere to endothelial biomarkers of interest, such as markers over-expressed in tumor angiogenesis [1,2].

Biomarkers specific to tumors could increase the sensitivity and specificity of traditional imaging techniques. In ultrasound molecular imaging, targeted microbubble contrast binds to endothelial markers directed by a targeting moiety such as an antibody or receptor ligand. Bound microbubbles, under ultrasound energy, then display detectable acoustic backscatter. Several potential applications of ultrasound molecular imaging exist. Molecular imaging of tumor endothelial markers may help better discriminate between benign and malignant disease [3,4]. After tumor treatment, molecular imaging may provide early indication of tumor response, as molecular changes may precede changes in tumor volume [2,5].

Ultrasound has several advantages over other imaging modalities such as MRI and CT. MRI is expensive, slow, and not widely available in rural locations. The cost of portable ultrasound systems is very affordable—typically only 1/100th of a MRI system [6], and unlike CT scans, ultrasound does not require ionizing radiation, and clinically approved ultrasound contrast agents are well tolerated [7].

The development of strategies for monitoring the angiogenic process depends upon identifying targets with biosignatures unique to tumor angiogenesis. To discover proteins overexpressed in tumor vessels, we developed a novel method for immuno-laser capture microdissection coupled with RNA amplification and genome-wide gene expression to profile tumor vasculature cells from human breast tumors with comparison to normal breast samples [8]. In our analysis, we identified that secreted frizzle related protein 2 (SFRP2) mRNA was increased more than 6-fold in breast cancer endothelium. We validated localization and overexpression of SFRP2 in tumor endothelium at the protein level as shown by immunohistochemistry in breast, colon, pancreas, renal, prostate, hepatocellular carcinoma, and angiosarcoma [8]. We further discovered that SFRP2 is a novel stimulator of angiogenesis *in vivo* [9] and *in vitro*, and that antagonism of SFRP2 with a mouse monoclonal antibody inhibited triple negative breast carcinoma and angiosarcoma growth in mice [10].

We previously created an antibody-labeled contrast by conjugating biotinylated polyclonal SFRP2 antibodies to biotin-activated microbubble ultrasound contrast using a streptavidin bridge [11]. We demonstrated that ultrasound molecular imaging using SFRP2-targeted contrast increased the average video pixel intensity (VI) of angiosarcoma vessels by 2.2 ± 0.6 VI compared to the streptavidin control. However, background binding of our control contrast was higher than expected and we sought to optimize our method to improve specificity. In this study, we hypothesized that optimizations to our molecular imaging reagents, and protocol could significantly increase the average video pixel intensity of angiosarcoma vasculature imaged with SFRP2-targeted contrast agent. We optimized five design parameters: (1) we decreased non-specific binding by using NeutrAvidin^™^-activated microbubbles, and eliminated microbubble aggregation by using a maleimide-thiol linkage rather than using a streptavidin bridge between biotinylated antibodies and microbubbles, (2) we decreased non-specific binding further by targeting our control contrast with antibodies against chicken IgY, (3) we used time-intensity curves (TIC) from non-tumor regions of interest (ROI) to generate free-flowing- corrected TICs for both SFRP2-targeted, and IgY-targeted contrast, which allowed us to time our molecular imaging acquisition to occur when the ratio between SFRP2-specific and IgY-specific acoustic signals was at its maximum, (4) we varied the antibody-labeling index, and (5) we varied the dose of targeted microbubbles. We then applied these optimizations to determine the performance of our optimized SFRP2-targeted contrast in imaging angiosarcoma tumors in a murine allograft model.

## Material and methods

### Cell culture

Murine SVR angiosarcoma cells were acquired from American Type Culture Collection (ATCC, Manassas, VA). ATCC authenticates cell line identity; furthermore, SVR angiosarcoma cells tested negative for Ectromelia, EDIM, LCMV, LDEV, MHV, MNV, MPV, MVM, Mycoplasma, Polyoma, PVM, REO3, Sendai, TME GFVII using PCR analysis performed by Research Analytic Diagnostic Laboratory (Columbia, MO). Cells were cultured in high-glucose DMEM with 10% fetal bovine serum (Sigma Aldrich, St. Louis, MO) and incubated in a 37°C humidified room air atmosphere enriched with 5% CO2.

### Animal care and husbandry

All animal experiments were performed in accordance with the recommendations in the Guide for the Care and Use of Laboratory Animals of the National Institutes of Health. The Institutional Animal Care and Use Committee of the University of North Carolina at Chapel Hill (UNC-CH) approved this study.

The Division of Laboratory Animal Medicine (DLAM) veterinarians, veterinary technicians and laboratory technicians supervise the health care and humane use of all animals on the UNC-CH campus, and have been accredited by the Association for the Assessment and Accreditation of Laboratory Animal Care International (AAALAC) since 1973. As laboratory animal specialists they provide a comprehensive program of veterinary care, offer technical advice and assistance, and provide laboratory and pathology services needed for diagnostic and research purposes.

Veterinary technicians make rounds through all animal facilities on a regular basis to assess the health status of all animals. Animal husbandry services provided by DLAM include: housing and husbandry services employing standardized food, bedding, caging, and cage changing intervals, with daily inspections of all cages; sanitation of cages, equipment and animal rooms; environmental enrichment; consultation on animal care and use, breeding, species selection, species specific biology and health care, and testing of sentinel animals for viral and bacterial pathogens, as well as endo and ectoparasites.

Although not required during this study, if any animal had experienced pain or discomfort that could not be alleviated with standard analgesics, they would have been humanely euthanized with an overdose of inhaled anesthetic or carbon dioxide, followed by a physical means of euthanasia. All animals at the end of the study, and any animal with a tumor in excess of 1.5 cm diameter were humanely euthanized as described above.

### Establishment of SVR angiosarcoma allografts *in vivo*

Injecting 1 x 10^6^ SVR angiosarcoma cells subcutaneously in the right flank of six week-old nude mice created tumor allografts. Tumor measurements were obtained twice weekly using B-mode ultrasound on a Vevo2100 (Visual Sonics, Toronto, Ontario, Canada). Ultrasound-based tumor measurements were performed under isoflurane anesthesia. Molecular imaging experiments were performed when tumors reached 8 mm in maximal dimension, which typically occurred after two weeks of growth.

### Targeted ultrasound contrast agents were designed with NeutrAvidin^™^-activated microbubbles

**Fig 1:**
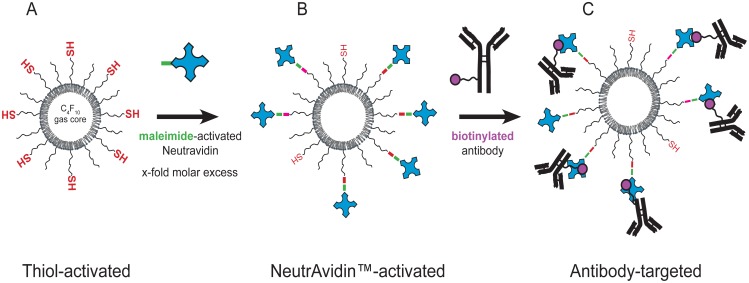
SFRP2-targeted and control ultrasound contrast agents were designed with NeutrAvidin^™^. **(A)** Sulfhydryl-activated lipid solution was formulated by incorporating a thiol functionalized PEG-lipid (DSPE-PEG2000-thiol) at 0.2 mole percent in our lipid solution. Thiol-activated contrast agent was prepared by shaking the lipid solution in 3 ml glass vials containing perfluorobutane gas (see Methods for details). **(B)** NeutrAvidin^™^ functionalized contrast agent was prepared by forming a covalent crosslink between maleimide-activated NeutrAvidin^™^ and thiol-activated contrast agent. We estimated the thiol content of our sulfhydryl-activated contrast agent and tested 3 and 10-fold molar excess of maleimide-activated NeutrAvidin^™^. **(C)** Anti- SFRP2 targeted or control anti-chicken IgY targeted microbubbles were prepared by adding a 3-fold molar excess of either biotinylated SFRP2 antibodies or biotinylated antibody to chicken IgY to NeutrAvidin^™^ functionalized, ultrasound contrast agents.

The amino moiety of 1,2-distearoyl-*sn*-glycero-3-phosphoethanolamine-N-[amino(poly-ethylene glycol)-2000] (DSPE-PEG2000-Amine) was converted to a sulfhydryl moiety using Traut’s reagent. The resulting sulfhydryl-activated lipid (DSPE-PEG2000-Thiol) was incorporated at 0.2 mole percent into a lipid solution containing 90 mole percent 1,2-distearoyl-sn-glycero-3-phosphocholine (DSPC), 9.8 mole percent 1,2-distearoyl-sn-glycero-3-phospho-ethanolamine-N-[methoxy(polyethylene glycol)-2000] (DSPE-PEG2000), 15% (v/v) propylene glycol, 5% (v/v) glycerol in phosphate buffered saline (PBS). Sulfhydryl-activated ultrasound contrast (Fig 1A) was prepared by shaking the lipid solution in glass vials containing decafluorobutane (DFB) gas using a VialMix (Lantheus Medical Imaging, N. Billerica, MA). Given that the head group of phosphatidylcholine occupied 0.71 square nm, we calculated that a 4 μm diameter microbubble could accommodate 1 × 10^8^ lipid molecules. The thiol content of our microbubble preparations were calculated based on the mole percentage of the DSPE-PEG2000-thiol, the average diameter and total number of microbubbles.

NeutrAvidin^™^ (Pierce Biotechnology, Rockford, IL) is a deglycosylated, neutral form of avidin with less nonspecific binding properties than native avidin. 3 or 10-fold molar excess of maleimide-activated NeutrAvidin^™^ was reacted with sulfhydryl-activated ultrasound contrast according to the manufacturer’s protocol to prepare NeutrAvidin^™^-coated microbubbles (Fig 1B). Unbound NeutrAvidin^™^ was removed by three sequential washes with PBS. NeutrAvidin^™^-coated microbubbles were stored at a concentration > 1 × 10^9^ microbubbles/ml at 4°C until needed for creation of targeted contrast.

Incubating biotinylated SFRP2 antibodies with NeutrAvidin^™^-coated microbubbles created SFRP2-targeted ultrasound contrast agent (Fig 1C). Three polyclonal antibodies (two raised in goat and one in rabbit) against different epitopes of SFRP2 were obtained from Santa Cruz Biotechnology (Santa Cruz, CA) sc-7426 (goat, C-18), sc-13940 (rabbit, H-140), and sc-31574 (goat, D-20). These antibodies were biotinylated using EZ-Link Sulfo-NHS-LC-Biotinylation Kit (Pierce Biotechnology) according to the manufacturer’s instructions. Unbound antibodies were removed by three sequential washes with PBS. SFRP2-targeted microbubbles were stored at a concentration of > 1 × 10^9^ microbubbles/ml at 4°C until needed for molecular imaging.

Incubating biotinylated polyclonal control antibodies with NeutrAvidin-coated microbubbles created control ultrasound contrast agent. Biotinylated polyclonal control antibodies were raised in either rabbit or goat against chicken IgY (Bethyl Laboratories, Montgomery, TX), and were used in the same 2:1 ratio of goat to rabbit as used with the SFRP2-targeted ultrasound contrast agent.

A typical size distribution for anti-SFRP2 targeted contrast or the control anti-chicken IgY targeted contrast was: 2.1±1.3 μm diameter, with a mode of 1.6 μm, and median diameter of 1.7 μm.

### Molecular imaging with SFRP2-targeted, and IgY-targeted control contrast agents

**Fig 2:**
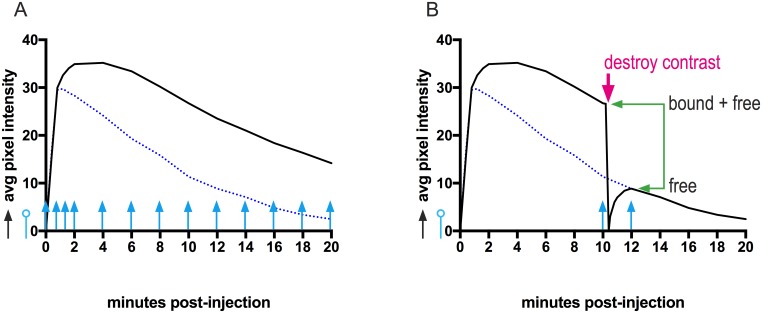
3-dimensional molecular imaging protocols estimated video intensity of free flowing contrast, with and without destruction of microbubbles. Prior to injection of targeted contrast agent, anatomical features were recorded with a B-mode scan (black arrow). The first contrast-specific, Cadence^™^ contrast pulse sequencing scan was performed in the absence of contrast (blue line capped with open circle) to establish the average video pixel intensity (VI) of ROIs at baseline. **(A)** Time-intensity curves (TICs). A ‘non-destructive imaging scheme’ captured multiple Cadence^™^ mode scans during the first 2 minutes post-injection to capture wash-in of the contrast bolus, and then every two minutes between 2 to 20 minutes post-injection. Average video pixel intensities from a representative study were plotted, showing total signal from tumor ROIs (black line) and from non-tumor ROIs (dotted blue line). The TIC from non-tumor ROIs was used later to approximate the signal contributed by freely flowing contrast. **(B)** An image—destroy—image’ scheme was used to differentiate targeted contrast bound within tumor endothelium from targeted contrast flowing freely in the vasculature. A Cadence^™^ mode scan was captured at a pre-determined time (10 minutes, pre-destruction scan, blue arrow) to determine the average video pixel intensity in the tumor ROI. This was followed immediately by a high mechanical index (D-Color) scan to destroy all bound and freely flowing contrast within the tumor ROI (red arrow, destructive scan). A pause of 60 seconds allowed freely flowing contrast to re-enter the tumor vasculature, and a final Cadence^™^ mode scan was captured (12 minutes, post-destruction scan, blue arrow) to estimate the average VI contributed by freely flowing contrast. The average video pixel intensity contributed by contrast bound to endothelium was calculated by subtracting the average VI of the post-destruction scan from the average VI of the pre-destruction scan. Average video pixel intensity for all scans was reported after subtracting the average video pixel intensity of the baseline scans.

Molecular imaging of SFRP2 expression was performed with the SFRP2-targeted and control contrast agents as previously described [11], and as illustrated in Fig 2. Briefly, animals were anesthetized with isoflurane and tail vein catheters were placed. Three dimensional ultrasound scans were captured using a Lab View (National Instruments, Austin, TX) controlled motion stage that coordinated transducer motion and image acquisition. Three-dimensional B-mode ultrasound images were obtained at 15 MHz using a 15L8 linear array transducer with a Siemens imaging system (Acuson Sequoia 512, Mountain View, CA). B-mode images allowed selecting the region of interest (ROI) in each imaging plane and allowed measuring tumor volume. Cadence^™^ pulse sequence (CPS) mode, a nondestructive contrast-specific imaging technique operating at 7 MHz (mechanical index = 0.18, CPS gain = –7 dB, dynamic range = 80 dB) was used to image targeted and control contrast agents.

#### Molecular imaging of SFRP2 expression with time-intensity curves

**Fig 3:**
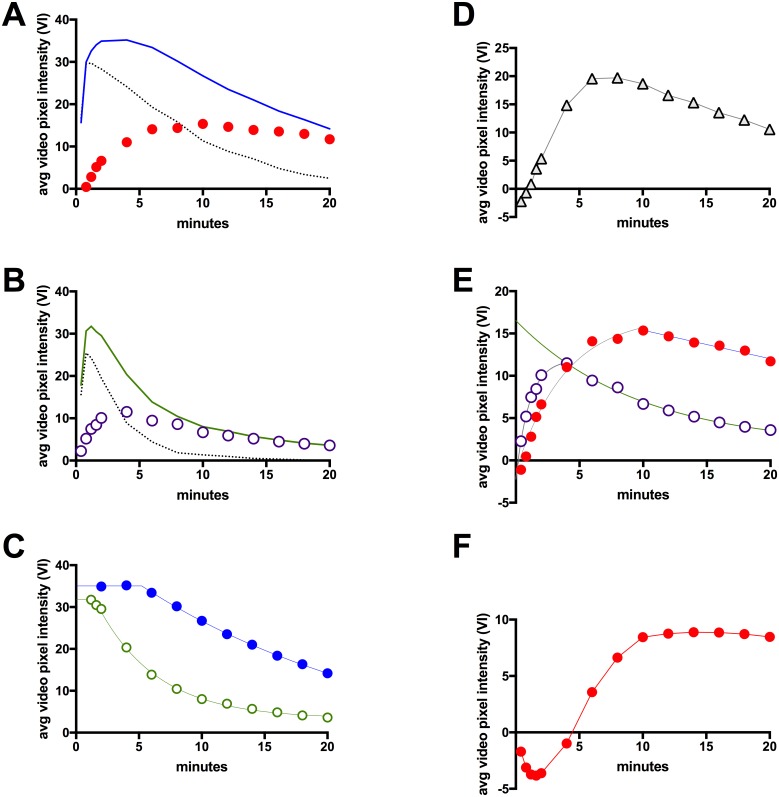
Time-Intensity Curves (TICs) generated from a representative animal that received both SFRP2-targeted, and IgY-targeted control contrast. **(A)** SFRP2-targeted contrast: solid blue line represents intensity within the tumor ROI, dotted black line represents intensity within a non-tumor ROI, red filled circles represent the difference between the tumor and non-tumor ROI intensities, which we term ‘free-flowing-corrected’ TIC (*ffc*-TIC). **(B)** IgY-targeted contrast: solid green line represents intensity within the tumor ROI, dotted black line represents intensity within a non-tumor ROI, purple open circles represent the difference between the tumor and non- tumor ROI intensities, which we term ‘free-flowing-corrected’ TIC (*ffc*-TIC). **(C)** The wash out of contrast from tumor ROI was modeled by one-phase exponential decay for IgY-targeted contrast (raw data, open green circles; green line, best-fit model), and by a plateau followed by one-phase exponential decay for SFRP2-targeted contrast (raw data, solid blue circles; blue line, best-fit model). **(D)** The difference between the models depicted in panel (C) was plotted with open black triangles. Note the maxima between 6–10 minutes. **(E)** The *ffc*-TICs for SFRP2, and IgY-targeted contrast were fitted to curves. A one-phase association model (grey line) fit the wash in portion of the *ffc*-TIC for SFRP2-targeted contrast (red filled circles), and for IgY-targeted contrast (open purple circles). The wash out of SFRP2-targeted *ffc*-TIC was best fit with a linear regression model (blue line), while the wash out of IgY-targeted *ffc*-TIC was best fit with a one-phase exponential decay model (green line). **(F)** The best-fit model for the IgY-targeted *ffc*-TIC was subtracted from the best-fit model for SFRP2-targeted *ffc*-TIC, and was plotted (red filled circles with red line). This produced a TIC representing the signal intensity within the tumor ROI that could be attributed to binding of contrast specifically mediated by the SFRP2 antibodies used to formulate the SFRP2-targeted contrast. N = 5 animals.

The average video pixel intensity within our 3D ROIs was used to plot time-intensity curves (TICs) to better understand the natural decay of contrast within the tumor after bolus injection (Figs 2 and 3). Cadence^™^ mode images were captured immediately after bolus injection of contrast agent, then every 30 seconds for 2 minutes (to establish peak), followed by every 2 minutes for 20 minutes (to establish washout). ROIs were defined using the anatomical features of the B-mode image with occasional guidance from the contrast-specific, Cadence^™^ image. The non-tumor ROI was drawn in a similar fashion, at approximately the same tissue depth as the tumor, avoiding any signal artifacts present on the baseline CPS image. The ROIs were not matched in pixel size since the metric for comparison was the average video pixel intensity. The peri-tumoral region was used to approximate the signal from free-flowing contrast present in the vasculature with the assumption that no contrast was bound outside of the tumor ROI. Average video pixel intensities for the tumor, and peri-tumoral regions were calculated for each contrast at every time point, and the best-fit model for signal decay was determined using GraphPad Prism. We also calculated a ‘free-flowing-corrected’ TIC for SFRP2 or IgY-targeted contrast by subtracting the relevant peri-tumoral average pixel intensity (Fig 3A, 3B and 3E).

To determine the optimal time for image acquisition after contrast bolus, we compared TICs for SFRP2-targeted contrast to control contrast. Five animals received a control contrast bolus followed by SFRP2-targeted contrast, under one episode of anesthesia. The greatest difference between the ‘free-flowing-corrected’ TICs for SFRP2-targeted and IgY-control contrast began to plateau at 10 minutes, and this time point was selected as the optimal time to acquire data for molecular imaging (Fig 3E and 3F).

#### Molecular imaging of SFRP2 expression using the ‘image—destroy—image’ method to subtract the contribution of free flowing contrast

Molecular imaging of SFRP2 expression was performed with the SFRP2-targeted and control contrast agents as previously described [11], as illustrated in Fig 2B.

Fifty μl of microbubble contrast was injected into the tail vein. At the specified time after bolus injection (discussed below), Cadence^™^ mode 3D scans of the tumor and surrounding tissue were performed to determine the average video pixel intensity contributed by microbubble contrast that remained within the tumor and surrounding tissue, and was referred to as the ‘pre-destruction’ scan. The Acuson Sequoia was preconfigured with a ‘D color’ scan setting that provided a short burst of high mechanical index ultrasound that destroyed microbubbles present in the field of view. After using high-energy D color scans to destroy all freely flowing, and all bound contrast agents within the scan volume, a second 3D Cadence^™^ mode scan was acquired, and was referred to as the ‘post-destruction’ scan. This final scan established the video pixel intensity attributable to free-flowing contrast remaining in the vasculature. The amount of contrast bound within any ROI was determined by subtracting the average post-destruction video pixel intensity from the average pre-destruction pixel intensity (Fig 2B). Average video pixel intensity for all scans was reported after subtracting the average video pixel intensity of the baseline scans.

### Optimizing antibody-loading of targeted contrast agents by increasing NeutrAvidin^™^-labeling of microbubbles

We created two formulations of SFRP2-targeted microbubble contrast to improve contrast binding to tumor. Sulfhydryl-activated microbubbles (0.2 mole percent) were incubated in either 3- or 10-fold molar excess of maleimide-activated NeutrAvidin^™^. Biotinylated SFRP2 antibodies were then bound to NeutrAvidin^™^ microbubbles as described above. If sulfhydryl moieties were left unmodified after the reaction with 3-fold molar excess of NeutrAvidin^™^, higher antibody binding would be expected after using a 10-fold molar excess of NeutrAvidin^™^. Eight animals received 5 x 10^6^ microbubbles of both the 3- and 10-fold molar excess formulations and were imaged 10 minutes after bolus injection. Tumor and adjacent tissue perfusions using both formulations were compared in each animal.

### Optimizing contrast agent dose

To determine the optimal dose of SFRP2-targeted and control microbubble contrast, we created four formulations of contrast: 5 × 10^6^ per 50 μl and 5 × 10^7^ per 50 μl for both SFRP2-targeted and control contrast. Ten animals received doses of 5 × 10^6^ followed by 5 × 10^7^ of either SFRP2-targeted or control contrast. Contrast enhancement in tumor and adjacent tissue was compared in each animal using all contrast formulations.

### Testing optimized molecular imaging reagents, and protocol *in vivo*

To verify the performance of our optimized contrast agents, we performed molecular imaging of 10 animals with control contrast, followed by SFRP2-targeted contrast using the ‘image—destroy—image’ scheme illustrated in Fig 2B. Cadence^™^ mode images were captured 10 minutes after bolus injection to determine the amount of bound contrast. A final Cadence^™^ mode scan was captured, after destroying all contrast within the scan region, to detect free-flowing contrast. Contrast injection and molecular imaging were performed for both contrast agents under a single episode of anesthesia. The SFRP2-targeted contrast was injected 10 minutes after the completion of the control contrast scans to ensure adequate washout of control microbubbles. Retention of contrast agents in tumor and non-tumor tissue was compared in each animal for both contrast agents.

### The relationship between microbubble acoustic signal, and video pixel intensity

Microbubble signal in arbitrary acoustic units (AU), spanned a dynamic range of 80 dB, and was log compressed to fit within an 8-bit range so that it could be represented visually. We calculated average video pixel intensities (VI) from these images, which served as a logarithm-based measure of changes in acoustic signal. To estimate linear changes in acoustic signal, it was necessary to log decompress average VI values using Eq 1 [12]:

$$\text{Acoustic signal}=\text{Linearized video signal}=255\times 10^{\left\{\frac{\left(VI-255\right)}{255}\times \frac{\text{dynamic range}}{20}\right\}}$$ (1)

**Fig 4:**
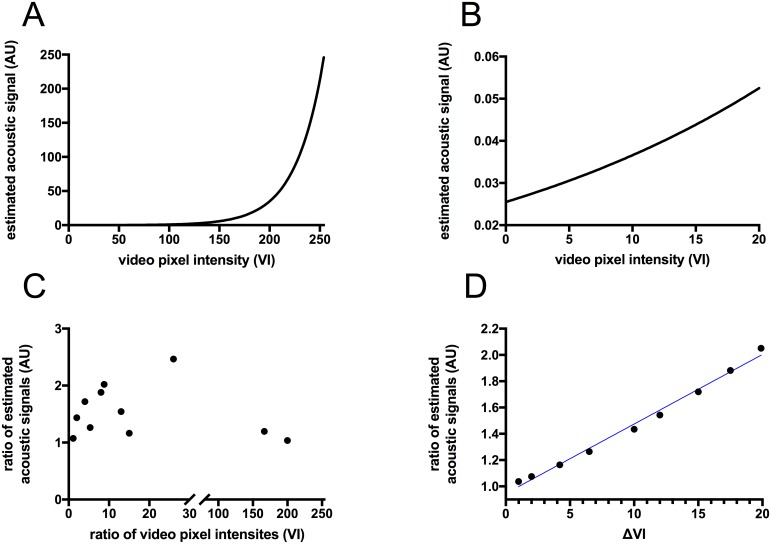
The relationship between microbubble acoustic signal, and log compressed video pixel intensity. **(A)** The acoustic signal from microbubbles in arbitrary acoustic units (AU) was log compressed from a dynamic range of 80 dB to an 8-bit range (0–255) of video pixel intensities (VI). Given video pixel intensities (VI), log decompression with Eq 1 was used to estimate the original acoustic signal in AU, and was plotted to show their non-linear relationship over the entire 8-bit range. **(B)** A linear relationship existed between video pixel intensities (VI), and their corresponding acoustic signals (AU), when VI was < 20. This linear relationship represented a range of acoustic signals limited to ~ 0.025–0.055 AU. **(C)** The ratio of any two video pixel intensities (VI) was not related linearly to the ratio of their estimated acoustic signals. (D) However, the ratio between log-decompressed acoustic signals (*AU*_**2**_ ÷ *AU*_**1**_) was related linearly to Δ VI = (VI2 –VI1). Computing ΔVI resulted in a metric that was proportional to the ratio of the original acoustic signals: a natural consequence of the Law of Exponents. Linear regression provided the best-fit equation: (*AU*_**2**_ ÷ *AU*_**1**_) = 0.0529 × Δ*VI* + 0.9455, with R^**2**^ = 0.992 describing the relationship between ΔVI, and (*AU*_**2**_ ÷ *AU*_**1**_) when ΔVI < 20.

Substitution into Eq 1 showed that the ratio of any two acoustic signals estimated from their average video pixel intensities (VI) was proportional to a power function with an exponent of ΔVI = (VI_2_ –VI_1_). From the Law of Exponents, the ratio of any two acoustic signals was proportional only to ΔVI, and could not be calculated by making a ratio between the two VI values (Fig 4). Log decompression estimated the original acoustic signal since ultrasound scanners may perform additional proprietary transforms of acoustic data before displaying the video data.

### Statistics

Custom MATLAB (Mathworks, Natick, MA) scripts were used to determine baseline- subtracted, average video pixel intensity (VI) of tumor and adjacent tissue using video data from DICOM files. Two-tailed, paired or unpaired t-test, were used to compare groups in the optimization experiments, as appropriate. Curves were fit to data, and plotted with GraphPad Prism (version 7.0a for Mac OS X, GraphPad Software, La Jolla CA, USA, www.graphpad.com). A p-value of 0.05 was considered significant. Statistical analyses were performed using SPSS (version 20, IBM, Armonk, New York) or GraphPad Prism.

## Results

### Molecular imaging of SFRP2 with time-intensity curves

Fig 3 depicts TICs for a representative animal receiving both SFRP2-targeted and control contrasts. We observed similar, rapid wash in peaks for both the SFRP2-targeted and control contrast agents, and relatively fast wash out of the contrast agents, which was expected given the short half-life of microbubble contrast (IgY-targeted T½ = 3.0 minutes, 95% CI = 2.8–3.3 minutes). Slower wash out occurred using the SFRP2-targeted contrast, secondary to SFRP2 binding to tumor vasculature (SFRP2 T½ = 17.3 minutes, 95% CI = 12.1–30.0 minutes). There were no differences in peak signal intensity between the SFRP2-targeted and control contrast groups.

The wash out of IgY-targeted contrast was best fit with a one-phase decay model where: Y = signal intensity, Y_0_ = signal intensity at time zero, x = time, k = rate constant, and Plateau = signal intensity at infinite time (Fig 3C).

$$Y=Plateau+\left(Y_{0}-Plateau\right)\cdot e^{\left(-kx\right)}$$ (2)

However, the washout of SFRP2-targeted contrast (Fig 3C) was best fit with a model using a plateau followed by one-phase decay where: x = time, x_0_ = time when decay begins, and remaining variables were as defined for Eq 2.

$$Y=Plateau+\left(Y_{0}-Plateau\right)\cdot e^{\left(-k\cdot \left[x-x_{0}\right]\right)}$$ (3)

Constants for the best-fit curves representing wash out of contrast from the tumor ROI (Eqs 2 and 3) are presented in Table 1.

**Table 1:** Best-fit values described nonlinear curves for wash out of contrast.

Contrast	SFRP2 targeted		IgY targeted	
ROI	Tumor		Tumor	
Curve	Plateau followed by one phase decay		One phase decay	
Constant	Value ± SE	95% CI	Value ± SE	95% CI
x_0_	3.28 ± 0.223	2.72 to 3.75	n/a	n/a
Y_0_	31.9 ± 0.205	31.4 to 32.3	39.5 ± 0.524	38.3 to 40.7
Plateau	-15.1 ± 6.37	-39.7 to -4.97	3.61 ± 0.232	3.07 to 4.12
x	0.0401 ± 0.00768	0.0231 to 0.0573	0.23 ± 0.0076	0.213 to 0.248
Half-life	17.3	12.1 to 30.0	3.01	2.80 to 3.25

The wash out of contrast from non-tumor ROIs was modeled using a one-phase decay. Coefficients for the best-fit models describing wash out of contrast from non-tumor ROIs are presented in Table 2.

**Table 2:** Best-fit values described models for wash out of contrast from non-tumor ROI.

Contrast	SFRP2 targeted		IgY targeted	
ROI	Non-tumor		Non-tumor	
Curve	One phase decay		One phase decay	
Constant	Value ± SE	95% CI	Value ± SE	95% CI
Y_0_	33.8 ± 0.539	32.7 to 35.1	35.1 ± 1.24	35.5 to 37.9
Plateau	-7.40 ± 2.14	-13.3 to -3.47	-0.0792 ± 0.406	-0.994 to 0.781
k	0.0746 ± 0.00768	0.0580 to 0.0918	0.322 ± 0.0240	0.275 to 0.376
Half-life	9.29	7.55 to 11.9	2.15	1.84 to 2.52

#### The maximum difference between SFRP2-targeted, and IgY-control TICs occurred before the IgY-control TIC reached minimal levels

Among the 5 animals that received both contrasts, the maximal difference between SFRP2-targeted and control contrast agents occurred between 4 and 12 minutes after injection. For the animal depicted, the maximal difference between the best-fit models for the SFRP2, and IgY control contrasts occurred between 6–8 minutes post-injection (Fig 3D). However, it required 10 minutes for the IgY-control TIC’s signal-to-noise ratio to fall to minimal levels (Fig 3C). We let the average video pixel intensity of the IgY-control TIC at infinite time (plateau) to represent the ‘noise’ level (Table 1, 3.6 ± 0.2 VI); ‘minimal’ was defined as a signal-to-noise ratio < 2.

#### Non-tumor ROIs were used to define ‘Free-Flowing-*Corrected’* TICs (*ffc-*TICs)

We estimated the amount of signal contributed by ‘free-flowing’ contrast by assuming that no contrast was bound by non-tumor tissue, and plotting the TIC of non-tumor ROIs (dotted lines in Fig 3A and 3B). By subtracting the ‘free-flowing’ TIC from the tumor TIC, we established ‘free-flowing-*corrected’* TICs (*ffc*-TICs) for SFRP2 and *ffc*-TICs for IgY-targeted contrast (Fig 3A, 3B and 3E). As expected, the wash in of the *ffc*-TICs fit a model of pseudo-first order kinetics for one-phase association (Fig 3E). The wash out of the SFRP2-targeted *ffc*-TIC was best fit with a straight line. However, the wash out of the IgY-targeted *ffc*-TIC was best fit with a one-phase exponential decay (Table 3). Peak intensity of the IgY-targeted *ffc*-TIC occurred 4 minutes after injection (11.5 VI) compared to the 10 minutes required for the SFRP2-targeted *ffc*-TIC to come to a slightly higher maximum (15.4 VI).

**Table 3:** Best-fit values described models for *ffc-*TICs.

Contrast	SFRP2 targeted		IgY targeted		
ROI	Tumor		Tumor		
Curve	Linear		One phase decay		
Constant	Value ± SE	95% CI	Constant	Value ± SE	95% CI
Slope	-0.337 ± 0.029	-0.417 to -0.257	Y_0_	16.5 ± 0.782	14.8 to 18.63
Y-intercept	18.8 ± 0.443	17.5 to 20.0	Plateau	1.68 ± 0.676	-0.706 to 2.946
X-intercept	55.6	47.8 to 68.2	k	0.104 ± 0.0146	0.0690 to 0.140
r^2^	0.9717		Half-life	6.685	4.94 to 10.0

#### The plateau in the control-subtracted, SFRP2-specific, *ffc*-TIC coincided with the optimal time to deploy the ‘image—destroy—image’ sequence for molecular imaging of SFRP2

The IgY-targeted *ffc*-TIC represented binding of contrast within the tumors that was not mediated by our mixture of SFRP2 polyclonal antibodies. By subtracting the IgY-targeted *ffc*-TIC from the SFRP2-targeted *ffc*-TIC, we were left with the contrast signal that could be directly attributed to contrast retained in the tumor specifically by the SFRP2 antibodies. 10 minutes after injection, the IgY-control-subtracted, SFRP2-targeted *ffc*-TIC reached a plateau of ~ 8.8 VI (Fig 3F). 10 minutes post-injection also corresponded to the point when the *ffc-*TIC for SFRP2-targeted contrast reached its maximum (Fig 3E), and also when the IgY-targeted TIC (Fig 3C) fell below twice the ‘noise’ level (Table 1, ‘plateau’ represents VI at infinite time). Accordingly, we chose 10 minutes as the optimal time point to obtain targeted molecular images for the remaining experiments.

### Increased NeutrAvidin^™^-labeling of microbubbles increased contrast enhancement

**Fig 5:**
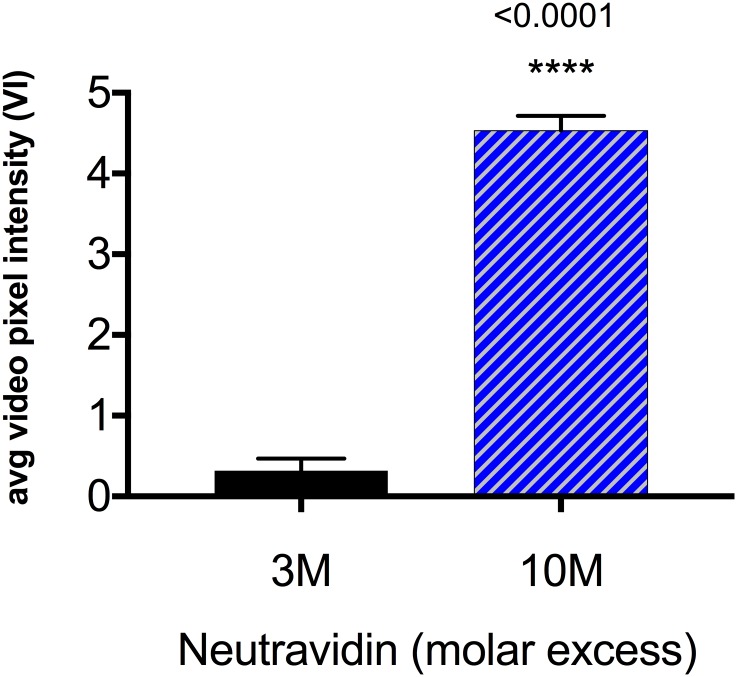
Average video pixel intensity increased with higher levels of NeutrAvidin^™^ labeling. Significantly higher tumor video pixel intensity was observed when SFRP2-targeted contrast was created using 10-fold molar excess of maleimide-activated NeutrAvidin^™^ compared to 3-fold molar excess. N = 8 animals.

SFRP2-targeted contrast created using 10-fold molar excess of maleimide-activated NeutrAvidin^™^ detected tumor vasculature with greater average video pixel intensity (4.53 ± 0.185 vs. 0.316 ± 0.150 VI, P<0.001) compared to control contrast formulated with 3-fold molar excess (Fig 5). Greater video intensity likely occurred secondary to higher antibody conjugation to the microbubbles, which therefore facilitated more frequent antibody-SFRP2 interactions within the tumor vasculature. There were no significant differences in signal intensity within the peri-tumoral tissue (not shown).

#### Labeling with maleimide-activated NeutrAvidin^™^ was nearly saturated

The change in VI from 0.32 to 4.53 VI represented a Δ VI of 4.2 for a dose of 5 × 10^6^ microbubbles. Since video pixel intensity (VI) represented microbubble acoustic signal in arbitrary acoustic units (AU) after log compression, Δ VI of 4.2 represented a 1.15 fold increase in the original microbubble acoustic signal (AU) after log decompression.

Thus, a 333% change in maleimide-activated NeutrAvidin^™^ (from 3 to 10-fold molar excess) only resulted in a 15% change in microbubble acoustic signal. This small increase in microbubble acoustic signal (AU) indicated that the maleimide moiety was near saturation for microbubbles formulated with a sulfhydryl content of 0.2 mole percent.

We considered testing a 100-fold molar excess of maleimide-activated NeutrAvidin^™^ but reasoned that the increase in linearized video signal might not match the previous 15% change. Increasing the molar excess of the maleimide moiety from 10 to 100-fold would have required increasing maleimide-activated NeutrAvidin^™^ from 500 mg per 1 × 10^10^ microbubbles to 5 mg per 1 × 10^10^ microbubbles; the cost to benefit ratio seemed high. In addition, since maleimide-activated NeutrAvidin^™^ was activated with an average of 4 moles of maleimide per mole of NeutrAvidin^™^, it was multivalent for sulfhydryl moieties making microbubble aggregation a real possibility at higher concentrations. Assuming the increase from 10 to 100-fold molar excess resulted in an additional 10% increase in microbubble acoustic signal, the total increase from 3-fold molar excess would have been 1.26 fold. This would have represented an increase in VI from 0.32 to 6.32, equivalent to a 26% increase in microbubble signal for a 33,000% increase in maleimide-activated NeutrAvidin^™^, which was an extremely high cost to benefit ratio.

Thus, we reasoned that 3 and 10-fold molar excess of maleimide-activated NeutrAvidin^™^ represented reasonable minimum, and maximum values respectively for the covalent attachment of maleimide-activated NeutrAvidin^™^ to 0.2 mole percent sulfhydryl-activated microbubble contrast.

### Optimizing contrast agent dose

**Fig 6:**
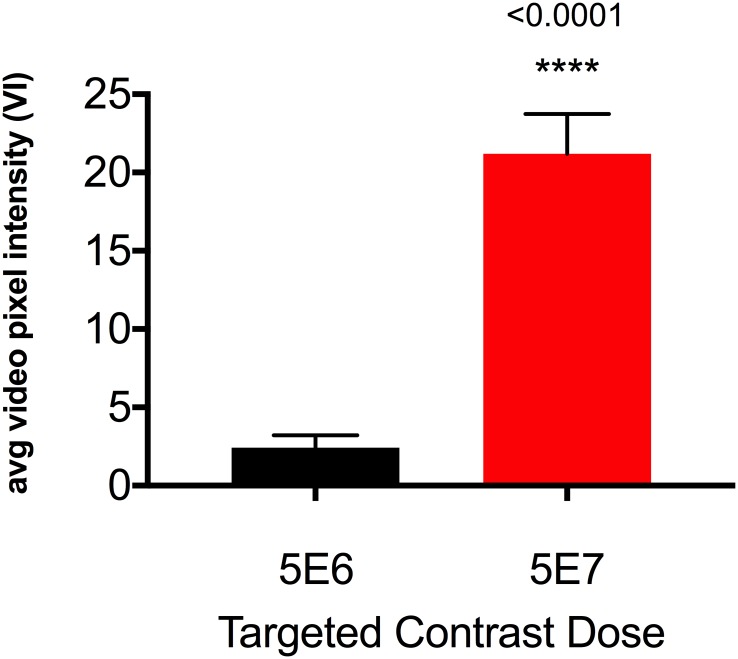
Average video pixel intensity increased with increased microbubble dose. Significantly higher video pixel intensity was observed in tumors when animals received 5 × 10^7^ SFRP2-targeted microbubbles compared to 5 × 10^6^ SFRFP2-targeted microbubbles. N = 10 animals.

Dose optimization was performed with SFRP2-targeted contrast formulated using 10-fold molar excess of maleimide-activated NeutrAvidin. SFRP2-targeted contrast injected as a bolus of 5 × 10^7^ microbubbles detected tumor vasculature with greater signal intensity compared to a bolus of 5 × 10^6^ microbubbles (Fig 6, 21.2 ± 2.53 vs. 4.53 ± 0.185 VI, p<0.001). There were no significant differences in signal intensity of peri-tumoral tissue, and peri-tumoral tissue retained significantly less contrast than tumor tissue (not shown).

#### Contrast agent dose was approaching saturation

Increasing the dose of targeted microbubbles from 5 × 10^6^ to 5 × 10^7^ resulted in a change in VI from 4.5 to 21.2 VI, a Δ VI of 16.7. This Δ VI corresponded to ~ 83% increase in acoustic signal for a 1,000% increase in microbubble dose. Although the percentage increase observed after changing microbubble dose was more robust than what was seen for changing antibody labeling index, we expected approximately 10-fold higher change if the microbubble dose was not near saturation. We concluded that further increases in microbubble dose might have provided a limited increase in acoustic signal.

However, the recommended dosing of Definity^®^ Perflutren Lipid Microspheres for human echocardiograms was 10 μL per kg of body weight. Assuming a patient mass of 154 lb. = 70 kg, a typical blood volume of 4.7 liters, and a maximum microbubble concentration of 1.2 × 10^10^ per ml, the recommended bolus dose of 0.7 ml provided 8.4 × 10^9^ microbubbles or 1.8 × 10^6^ microbubbles per ml of blood. Our nude mice weighed approximately 25 g, and were expected to have a total blood volume of 2.3 ml [13]. Bolus doses of 5 × 10^6^, and 5 × 10^7^ microbubbles resulted in 2.2 × 10^6^, and 22 × 10^6^ microbubbles per ml of blood, approximately 1.2 × and 12 × the recommended human bolus dose.

A higher contrast agent dose of 5 × 10^8^ was considered but was expected to have several drawbacks. First, the time required for the majority of freely flowing contrast to clear from circulation was expected to increase dramatically, compared to a dose of 5 × 10^7^ microbubbles.

The more important consideration was that 5 × 10^8^ microbubbles represented ~220 × 10^6^ microbubbles per ml of blood, equivalent to ~120 × the recommended human bolus dose. A lower dose of 5 × 10^5^ microbubbles was also considered but was rejected since lower signal intensity was not our goal. Accordingly, we decided that 5 × 10^6^, and 5 × 10^7^ represented the minimum, and maximum doses respectively of targeted microbubbles for molecular imaging of SFRP2 expression in angiosarcoma.

### Significantly improved performance of optimized molecular imaging reagents, and protocol verified *in vivo*

**Fig 7:**
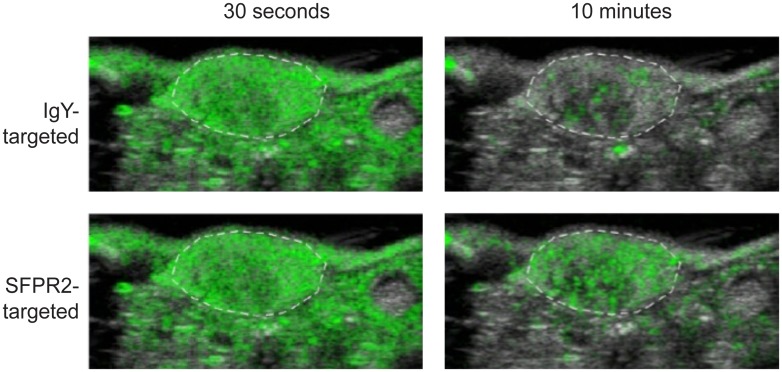
Ultrasound molecular imaging of animal receiving SFRP2-targeted and control IgY-targeted contrast. A white dashed line outlines tumors. The contrast-specific signal (green) was superimposed over the b-mode image (grey). At 30 seconds, average video pixel intensity was similar between control and SFRP2-targeted contrast. The contrast-specific video intensity was retained in tumors at much higher levels when using the SFRP2-targeted contrast compared to the IgY-targeted contrast.

**Fig 8:**
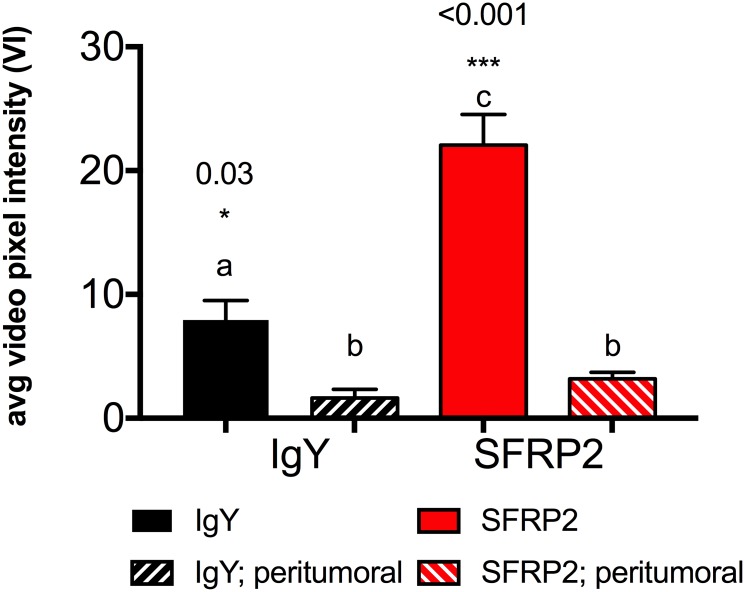
Verifying performance of optimized molecular imaging reagents, and protocol *in vivo*. Non-identical superscripts indicate a statistically significant difference in means. Both IgY-targeted (a, p = 0.03), and SFRP2-targeted (c, p < 0.001) contrast produced significantly higher video pixel intensities in tumor ROI compared to peri-tumoral regions (b). SFRP-2 targeted contrast (c, p < 0.001) produced significantly higher signal intensity in tumor ROI than the control IgY-targeted contrast (a). Average video pixel intensities were corrected for free flowing contrast by subtracting the post-destruction VI from the pre-destruction VI as shown in Fig 2B for the ‘image—destroy—image’ method. N = 10 animals.

SFRP2-targeted and IgY-targeted control contrast agents were created using 10-fold molar excess of maleimide-activated NeutrAvidin, and delivered in bolus doses of 5 × 10^7^ microbubbles per 50 μl. Molecular imaging data was captured 10 minutes after bolus injection using the ‘image—destroy—image’ scheme (Fig 2B). Cadence mode images from a representative animal illustrated the wash in and preferential retention of SFRP2-targeted contrast in tumor compared to the control IgY-targeted contrast (Fig 7). Using these optimizations, SFRP2-targeted contrast detected 14.2 ± 3.0 VI greater average video pixel intensity compared to the IgY-control in tumor vasculature (Fig 8: 22.1 ± 2.5 vs. 7.9 ± 1.6 VI, p <0.001). The specificity of our SFRP2-targeted contrast for tumor vasculature was confirmed by the minimal signal intensity for both the SFRP2 and control contrast agents in peri-tumoral vasculature (3.2 ± 0.52 vs. 1.6 ± 0.71 VI, p = 0.16).

When the IgY-targeted average video pixel intensity was subtracted from the SFRP2-targeted average video pixel intensity, we were left with a value of 14.2 ± 3.0 VI for the tumor. This value for control-subtracted, SFRP2-targeted video intensity can be attributed solely to the SFRP2 antibodies that mediated binding of contrast agent to tumor vasculature. These video intensities were corrected for the contribution of free-flowing contrast by destroying all bound contrast within the tumor volume, and then measuring the increase in video intensity caused by contrast agents carried back into the tumor by blood flow.

Half-lives for wash out of contrast from tumor ROIs and from non-tumor ROIs were calculated from the TICs (Tables 1 and 2). As expected, it took longer for SFRP2-targeted contrast to wash out from tumor vasculature than the IgY-targeted contrast (5.75 times longer; T½: 17.3 vs. 3.01 minutes; k: 0.0401 ± 0.00768 vs. 0.23 ± 0.0076). The wash out of SFRP2-targeted contrast from the tumor ROI was also 1.9 times slower than the wash out of SFRP2-targeted contrast from the non-tumor ROI (T½: 17.3 vs. 9.29 minutes; k: 0.0401 ± 0.00768 vs. 0.0746 ± 0.00768) indicating specificity for tumor vasculature. The wash out of IgY-targeted contrast from the tumor ROI was 1.4 times slower than its wash out from the non-tumor ROI (T½: 3.01 vs. 2.15 minutes; k: 0.23 ± 0.0076 vs. 0.322 ± 0.0240). As this ratio was > 1, we concluded that our IgY-targeted contrast was retained at a low level within tumor vasculature; this presumably represented nonspecific binding attributable to our microbubble phospholipid, NeutrAvidin^™^, and/or antibody composition.

Our previous publication compared biotinylated versions of the same polyclonal SFRP2 antibodies used in the current study, linked to biotinylated microbubbles by a streptavidin bridge versus control biotinylated microbubbles that were bound to streptavidin. The average video pixel intensity for SFRP2-targeted contrast reported in that publication was 2.17 ± 0.592 VI units higher (n = 13, p =  0.0032) than the streptavidin control [11]. Our improved ‘image—destroy—image’ scheme in conjunction with our improved SFRP2-targeted and the IgY-control contrast agents resulted in a significantly higher enhancement of average video pixel intensity (14.2 ± 3.0 VI, p = 0.0001). Log decompression of 2.17 Δ VI showed that streptavidin-linked SFRP2-targeted contrast produced ~ 4% higher acoustic signal (AU) than the streptavidin control. Log decompression of 14.2 Δ VI showed that NeutrAvidin^™^-linked SFRP2-targeted contrast produced ~ 70% higher acoustic signal (AU) than the NeutrAvidin^™^-linked IgY-targeted control. Our optimized contrast agents plus optimized molecular imaging protocol produced ~ 18-fold higher control-subtracted acoustic signal for SFRP2-targeted contrast compared to our previously published result.

## Discussion

Angiosarcoma is a highly lethal malignancy of vascular endothelial cells, and new strategies for molecular-based imaging are needed. We sought to improve ultrasound molecular imaging of secreted frizzled related protein 2, a secreted multi-function protein overexpressed in angiosarcoma endothelium [9]. We demonstrated improved performance of molecular imaging using simple optimizations including better timing of image acquisition and higher doses of contrast created using higher antibody labeling. Microbubble contrast is considered safe, and we observed no toxicity in animals receiving SFRP2-targeted or control contrast. SFRP2-targeted imaging provides potential advantages over traditional imaging such as enhanced tumor specificity and may yield information regarding tumor biology or response to therapy.

### Improved molecular imaging reagents, and protocols

Methodologically, multiple changes were made in our ultrasound molecular imaging technique, compared to previous studies. In this study, we omitted a microbubble size-sorting step, which reduced the time required to prepare different formulations of targeted and control contrast agents. Secondly, we changed the microbubble-antibody linking molecule from streptavidin to maleimide-activated NeutrAvidin^™^. Combining sulfhydryl-activated microbubbles with maleimide-activated NeutrAvidin^™^ reduced the formation of microbubble clusters, which occurred sporadically when bridging biotin-labeled microbubbles, and biotin-labeled antibodies with multivalent streptavidin. In addition, we expected the NeutrAvidin^™^ linking molecule to have less background binding in tissue, and cause fewer adverse reactions when used in humans. Although no toxicity studies or histopathology were performed, animals recovered fully from molecular imaging sessions with our NeutrAvidin^™^ based, antibody-targeted contrast, and experienced no more discomfort or distress than expected for receiving isoflurane anesthesia. Third, we directed the targeting of the control contrast with antibodies to chicken IgY to further reduce the non-specific binding seen when using streptavidin contrast as a control. Fourth, compared to previous studies, we sought to systematically identify the best timing for image acquisition after bolus injection of contrast agent.

Prior to this study, we chose to perform imaging at 18 minutes, as this appeared to be the time required for all free-flowing microbubbles to clear from the vasculature. Ideally, we wanted to know when the ratio of SFRP2-targeted contrast to IgY-targeted contrast had reached a maximum, after subtracting the contribution from freely flowing, unbound contrast. We used the average VI of a non-tumor ROI to approximate the signal returned by freely flowing contrast (*ffc*) for both anti-SFRP2 and IgY targeted contrast, and plotted the *ffc*-corrected TICs in Fig 3E. By subtracting the *ffc*-TIC for IgY-targeted contrast from the *ffc*-TIC for anti-SFRP2, we showed that the ratio between the ‘bound’ components of SFRP2-targeted and IgY-targeted contrast plateaued approximately 10 minutes post-injection (Fig 3F). We concluded that the optimal time to acquire molecular imaging data using the ‘image—destroy—image’ scheme was 10 minutes post-injection because this was when the IgY control-subtracted, *ffc*-TIC for SFRP2 reached its maximum (Fig 3F).

Taken altogether, these changes in methodology significantly improved the video pixel intensity of SFRP2-specific imaging of angiosarcoma tumors compared to the IgY-targeted control, and provided 18-fold higher acoustic signal from tumor-bound SFRP2-targeted contrast than our previous reagents, and protocol [11].

#### Linearizing Δ video pixel intensity simplified optimization

The optimization of crosslinking reactions or binding processes generally uses titration to determine when the reaction or binding is complete. Le Chatelier's principle allows these reactions to be driven by shifting the chemical equilibrium to counteract the effect of the excess reagent or increased concentration of a binding molecule such as an antibody. Empirically, the reaction is complete when it can no longer shift its equilibrium: eg. the inability to create more NeutrAvidin^™^-labeled microbubbles in response to increased concentration of maleimide-activated NeutrAvidin^™^ or no further binding of antibody-labeled microbubbles to tumor endothelium in response to increased input of the antibody-labeled microbubbles.

It is straightforward to assess how close a system is to saturation when inputs and outputs are measured on a linear scale. It is more problematic when inputs are varied on a linear scale, and outputs are measured on a log-compressed scale. Since microbubble acoustic signals from molecular imaging are generally on the very low end of our 80dB dynamic range, the log-compressed video pixel intensity scale has the effect of expanding small changes in the linear acoustic scale. In addition, since Δ VI is the unit that is proportional to the ratio of the acoustic signals, the relationship between video intensity and microbubble signal becomes even less intuitive.

When we expressed the changes (Δ VI) observed in our optimization experiments for NeutrAvidin^™^ labeling and for microbubble dose as linearized acoustic signals on an arbitrary log-decompressed scale, it was clear that our ‘high’ doses produced a very small percentage increase in the acoustic signal from microbubbles. It would have been possible to ‘optimize’ our conditions using only the log-compressed video intensity scale but it would have required many more doses to observe saturation, and it was likely that we would have chased diminishing returns by testing higher and higher doses. Log-decompression did not return the actual acoustic signal of microbubbles since the result was in arbitrary units, and because a variety of transforms can be applied to acoustic data before it is displayed on an 8-bit video scale but it was a useful tool to compare the effect of different treatments on a linearized scale.

#### Molecular imaging of SFRP2 using time-intensity curves

By examining the TICs obtained from a single animal (Fig 1E), we were able to estimate the video pixel intensity of freely flowing contrast within the tumor vasculature. Other groups have proposed methods to measure the amount of free-flowing contrast within vasculature directly [14,15]. We opted to use the average video pixel intensity in non-tumor ROIs to approximate the amount of free flowing contrast in the tumor, and corrected the average video pixel intensity of the tumor ROI accordingly. Using this method the amount of free-flowing-corrected (*ffc*) SFRP2-targeted contrast bound within the tumor was 15.4 VI for this animal. This value compared quite favorably to the difference between SFRP2-targeted video intensity, and IgY-control video intensities corrected for free flowing contrast by the traditional ‘image—destroy—image’ scheme illustrated in Fig 2B (Δ VI = 14.2 ± 3.0 VI, Fig 8).

Our TIC analysis also allowed us to examine the relationship between time, and the amount of tumor-bound signal directly attributable to the SFRP2 antibodies. Subtracting the IgY- targeted *ffc-*TIC from the SFRP2-targeted *ffc-*TIC removed any component of VI that could be attributed to non-specific binding: by the non-target antibody, the microbubble shell or the NeutrAvidin used to form the bridge between the two moieties. The calculated value for SFPR2 antibody-specific video intensity was 8.8 VI for this animal, which was slightly lower than the average control-subtracted value determined by the ‘image—destroy—image’ method (Fig 8).

The analyses of our TICs also systematically determined the optimal time for acquiring molecular imaging data when using the traditional ‘image—destroy—image’ sequence to subtract the contribution of free flowing contrast. In this study, the TICs determined that microbubbles washed out of the system much more quickly than our previous estimate, which was established by visually assessing free flowing contrast content in real-time, Cadence^™^ mode images (10 minutes versus 18 minutes). Other, more rigorous methods [12,16] have been proposed to differentiate between free flowing and bound contrast, however we feel that the analysis of tumor and non-tumor ROIs is straightforward, and simpler. Our method is valid as long as the assumption holds true that contrast does not bind in non-tumor tissue. As antibody-antigen binding kinetics might vary in other systems, we recommend performing TICs prior to validating untested tumors or targeted contrast formulations.

#### Clinical applications

In a clinical setting, it is not likely that a control targeted-microbubble would be deployed, and the clinician would not have the luxury of determining the optimal time to employ the traditional ‘image—destroy—image’ scheme (Fig 2B). However, if TIC data were collected in the same molecular imaging session that terminated with the traditional ‘image—destroy—image’ sequence, clinicians would be able to establish whether the amount of targeted-microbubble retained within the tumor had reached a stable level by the time of the final image acquisition by simply plotting the free-flowing-corrected time-intensity curves. This hybrid approach to molecular imaging would provide a metric establishing the amount of video contrast enhancement in tumor vasculature versus non-tumor tissue; it would be especially useful if the half-life of targeted contrast agents changed as a consequence of patient immune response [17,18].

Our study represents one of many possible applications of ultrasound molecular imaging. Celebi et al recently described the assessment of ischemia reperfusion injury using selectin-targeted microbubbles in a mouse model of testicular torsion [19]. They showed increased microbubble signal in animals receiving torsion, which was even higher in animals receiving torsion and TNF-α, compared to control. There were no perfusion differences in animals receiving control contrast. In our laboratory, Shelton *et al* demonstrated high-resolution super- harmonic ultrasound molecular imaging using avβ3 integrin in a rat fibrosarcoma model [20]. They demonstrated high-resolution 3D volumes of micro vascular anatomy, and when combined with ultrasound-based acoustic angiography, new opportunities for analyzing relationships between micro vascular anatomy and endothelial targets. Zhang et al used microbubbles conjugated to two neuropilin-1 targeted antibodies to image tumor angiogenesis in a murine model of breast cancer [21]. Further possibilities include molecularly targeted drug or gene delivery, or high-energy ultrasound tumor ablation using microbubbles that target endothelial biomarkers.

Any immune response generated to targeted microbubble contrast would be undesired: this would reduce the amount of circulating contrast agent, and could induce adverse reactions such as anaphylaxis. Thus, translation of SFRP2 targeted contrast to human applications will require using a humanized antibody, preferably a Fab-fragment that lacks the Fc region, to minimize immunogenicity, as well as the use of a direct, covalent linkage to the microbubble shell to eliminate the use of NeutAvidin^™^, another possible trigger for an immune response.

Ultrasound molecular imaging of SFRP2 represents a novel approach to visualize angiosarcoma vessels non-invasively. Optimizations to SFRP2 molecular imaging appear to increase the specificity for tumor vasculature, and may provide new opportunities for targeted molecular imaging. We expect that our simple optimizations could be applied to most antibody-linked microbubble ultrasound imaging systems to improve specificity and performance.
